# Impact of Body Composition on Prognosis and Dose-Limiting Toxicities on Metastatic Colorectal Cancer

**DOI:** 10.3389/fnut.2021.671547

**Published:** 2022-01-27

**Authors:** David da Silva Dias, Mafalda Machado, Carolina Trabulo, Beatriz Gosálbez, Paula Ravasco

**Affiliations:** ^1^Medical Oncology Department, Centro Hospitalar Universitário do Algarve, Faro, Portugal; ^2^Centre for Interdisciplinary Research in Health, Universidade Católica Portuguesa, Lisbon, Portugal; ^3^Faculdade de Ciências da Saúde da Universidade da Beira Interior, Covilhã, Portugal; ^4^Radiology Department, Centro Hospitalar Universitário do Algarve, Faro, Portugal; ^5^Medical Oncology Department, Centro Hospitalar Barreiro Montijo, Barreiro, Portugal; ^6^Católica Medical School, Universidade Católica Portuguesa, Lisbon, Portugal; ^7^Centro de Investigação Interdisciplinar Egas Moniz (CiiEM), Instituto Universitário Egas Moniz (IUEM), Caparica, Portugal

**Keywords:** body composition, body mass index, skeletal muscle index, sarcopenia, metastatic colorectal cancer, dose limiting toxicities, neutrophil-to-lymphocyte ratio, systemic inflammation

## Abstract

Sarcopenia is a progressive skeletal muscle disease, often present in oncological patients, that is associated with multiple adverse events such as worse prognosis, physical performance, and quality of life. Body composition evaluation by CT cross-section at the L3 vertebrae region appears to be a precise method to quantify skeletal muscle. The optimal cut-off for the definition of sarcopenia is not yet established, therefore the incidence of sarcopenia varies according to different studies. The main goal was to evaluate the presence of sarcopenia in patients with metastatic colorectal cancer (mCRC) and its impact on overall survival (OS) and dose-limiting toxicities (DLT). A retrospective cohort study of 178 patients with mCRC under first-line chemotherapy (ChT) in association with target therapy, in two hospital units, between January 2015 and December 2018. Skeletal mass area (SMA) was quantified with the *NIH ImageJ* software in CT cross-sectional images at the L3 vertebrae region. Statistical analysis was performed with *IBM SPSS v25 software* *https://www.ibm.com/analytics/spss-statistics-software*. The median age was 62 (SD ± 11) years old, 65% were men and 62.9% had an Eastern Cooperative Oncology Group (ECOG) performance status of 0. The cut-off value was established based on ROC analysis, with sarcopenia defined as SMI < 49.12 cm^2^/m^2^ for men and < 35.85 cm^2^/m^2^ for women. Despite the mean body mass index (BMI) of 25.71 (± 4.71) kg/m^2^, half of the patients presented sarcopenia. In a multivariate analysis using a Cox regression model, an association was observed between OS and higher ECOG PS (*p* = 0.014; HR 5.46, CI 95% [1.42–21.10]), neutrophil-to-lymphocyte ratio (NLR) >2.80 (*p* = 0.038; HR 2.20, CI 95% [1,05–4.62]), and sarcopenia (*p* = 0.01; HR 4.73, CI 95% [1.85–12.09]). Additionally, in a logistic regression model, age (*p* = 0.014; OR 1.09, IC 95% [1.02–1.16]) and sarcopenia (*p*= 0.030, OR 4.13, IC 95% [1.15-14.8]) were associated with higher incidence of DLT. The CT evaluation of the body composition at the L3 region allows for the quantification of sarcopenia, providing prognostic information and predictive value of DLT in patients with mCRC, although the establishment of optimal cut-off values are required for implementation in clinical practice. A multimodal strategy to delay muscle waste should be considered in these patients.

## Introduction

Colorectal cancer is the second most diagnosed cancer in Europe with 499,000 new cases in 2018 and the second with the highest mortality accounting for approximately 242,000 deaths (1). In the last two decades, the incidence has been increasing, meanwhile, patients with metastatic colorectal cancer (mCRC) have presented improvements in survival, possibly explained by the creation of multidisciplinary teams to provide the best therapeutic decision, as well as the new approaches to oligometastatic disease and new systemic target therapies (2).

Body composition can be evaluated by several methods such as anthropometric measurements as well as electric bioimpedance analysis (BIA). Although these are non-invasive, inexpensive, and reproducible, they lack the precision of other methods such as *dual-energy x-ray absorptiometry* (DXA), CT, and MRI (3). DXA has great accuracy to detect body composition, however, it is not used routinely in oncologic patients. CT scan has been a promising and reliable method to detect body composition in patients with cancer, with no extra costs nor toxicities as it is already performed routinely at diagnosis and follow-up. It appears to be especially efficient at detecting the skeletal muscle area (SMA) at the cross-section of the L3 vertebrae region, allowing the quantification of muscle mass with great precision when correlated to DXA (4).

Patients with mCRC present sustained body weight (BW) loss and muscle wasting, explained by several factors such as insufficient caloric and protein intake, antineoplastic treatments, and metabolic alterations secondary to chronic systemic inflammation and neoplastic cachexia. These complex processes promote alterations in the body composition of oncologic patients, including sarcopenia (5). Sarcopenia derives from the Greek words for flesh (sarx) and loss (penia) and has been recently considered a disease on ICD-10-CM (M62.84) (6). It has been challenging to define sarcopenia, although many expert groups have addressed this issue such as the European Working Group on Sarcopenia in Older People (EWGSOP) (7, 8), International Working Group in Sarcopenia (9), and Asian Working Group for Sarcopenia (10). In the last consensus of EWGSOP, sarcopenia has been defined as a progressive and generalized skeletal muscle disorder associated with an increased likelihood of adverse outcomes including falls, fractures, physical disability, and mortality. For diagnosis purposes, sarcopenia is probable if low muscle strength is detected, although diagnosis is only confirmed with the presence of low muscle quantity or quality. Despite considering CT evaluation a useful method to quantify sarcopenia and expected to be more widely used in the future, no cut-off values were established (7). The incidence of sarcopenia in mCRC varies depending on the cut-off applied, race and gender, although it can be as high as 71% (11). Sarcopenia has been associated with reduced overall survival (12) and quality of life (13) as well as more toxicities (14), and increased costs for the institution and health system (15, 16). In the present study, the incidence of sarcopenia will be procured in this population with mCRC and analyzed whether CT-defined sarcopenia at the L3 level could predict overall survival (OS) and dose-limiting toxicities (DLT).

## Materials and Methods

### Patients and Procedures

Retrospective cohort study based on data collected in medical records of 178 patients with colorectal carcinoma confirmed histologically, stage IV, under first-line metastatic chemotherapy (ChT) in association with target therapy such as epidermal growth factor receptor inhibitor, panitumumab, or vascular endothelial growth factor inhibitor, bevacizumab. Patients were identified based on the Regional Oncological Registry (ROR) in two hospital units at Centro Hospitalar Universitário do Algarve (CHUA), in Portugal, between January 2015 and December 2018. This study was conducted according to the guidelines of the Declaration of Helsinki and was approved by the Ethics committee for health care of Centro Hospitalar Universitário do Algarve, with a waiver for informed consent (Protocol UAIF 85/2020, approved in 26.08.2020). The data collected included demographic variables (gender, age, comorbidities, performance status [PS], and anthropometric measurements such as BW and height), variables related to the tumor (colon or rectal cancer, colon laterality, RAS mutation status, organs affected by metastasis), related to inflammation (neutrophils and lymphocytes), and treatment (treatment protocol applied, metastasectomy and toxicities according to *common terminology criteria for adverse events—CTCEA v5.0*).

The CT scan images at diagnosis of the metastatic disease of 81 of the 178 patients were analyzed using the *National Institute of Health ImageJ* software https://imagej.nih.gov/ij/download.html by a single observer. SMA at the cross-sectional area of L3 region was identified and delineated by its anatomic features using the hardware *Wacom One* https://www.wacom.com/en-us/products/pen-tablets/one-by-wacom. The muscles included were *psoas major, quadratus lumborum, erector spinae, latissimus dorsi, abdominal oblique muscles*, and *rectus abdominis*. SMA was quantified using pre-established−29 to 150 Hounsfield units (HU) range for skeletal muscle and was expressed in square centimeters (cm^2^). The skeletal muscle index (SMI) was calculated by dividing the SMA by the square of the height and the results were reported as square centimeters per square meters (cm^2^/m^2^).

### Outcomes

The primary endpoint was OS, defined as the time between the beginning of the 1st line palliative treatment and the event of death. The secondary endpoint was DLT, defined as severe toxicities requiring dose reduction, delay, and/or discontinuation of oncological drugs under first-line treatment. Discontinuation of the drug due to disease progression was not accounted for DLT definition.

### Statistical Analysis

Statistical analysis was performed with *IBM SPSS Statistics v25* software. Treatment of missing data was based on the listwise deletion method. Continuous variables were reported as means and their standard deviation. Differences in means with normal distribution were analyzed with the *t*-student test for two independent samples. Continuous variables that do not respond to parametric parameters were analyzed with *Mann-Whitney U-test*. For the association of categorical variables, the chi-square test (X^2^) was performed. The definition of cut-off points for sarcopenia (SMI) and systemic inflammation (NLR—Neutrophil-to-lymphocyte ratio) was obtained by *receiver operating characteristic* (ROC) analyses. Survival analyses were obtained by the *Kaplan-Meier* method with the *log-rank* test. Lastly, with the intention of reducing Type-I error, a multivariate analysis with Cox regression model was performed with variables that present association with OS on a univariate level, and a multivariate analysis with logistic regression model was performed on variables associated with DLT. The results were reported with a hazard ratio (HR) or odds ratio (OR) as a measure of association and confidence interval (CI) of 95%.

## Results

### Basal Characteristics

A total of 178 patients were analyzed, 65 underwent treatment with ChT plus panitumumab (ChT-Pan) and 113 with ChT plus bevacizumab (ChT-Bev). The mean age was 62 (SD ± 11) years old, 65.7% of patients were male and 62.9% of individuals had an ECOG PS of 0. The RAS mutation was present in at least 35%. The most common site of metastasis was the liver (77.5%) and metastasectomy on oligometastatic patients was performed in 21 patients (11.8%). The mean BW was 71.12 (SD ± 15.9) kg and the mean body mass index (BMI) was 25.71 (SD ± 4.71) kg/m^2^. SMI was evaluated in 45.5% (*n* = 81) of the population and sarcopenia was defined in this population of patients with mCRC as SMI < 49.12 cm^2^/m^2^ for men and < 35.85 cm^2^/m^2^ for women. Despite 61.1% of patients having normal weight or were overweight, and 18.5% were obese, sarcopenia was present in 49.4% of patients. Sarcopenic obesity, defined as the presence of sarcopenia and BMI ≥ 30 kg/m^2^, was observed in 3 patients. Basal characteristics of the population can be seen in greater detail in Table 1.

**Table 1:** Basal characteristics.

**Variable**	**Total *n* = 178**
**Age**	62.33 ± 10.57
**ECOG PS**	
0	112–62.9%
1	41–23.0%
≥2	25–14.1%
**Diabetes mellitus**	
0	156–87.6%
1	22–12.4%
**Laterality**	
Left colon	68–53.5%
Right colon	59–46.5%
**Metastasis (M1)**	
Liver	138–77.5%
Lung	51–28.7%
Peritoneum	35–19.7%
**Metastasectomy**	
0	157–88.2%
1	21–11.8%
**Treatment**	
Panitumumab	65–36.5%
Bevacizumab	113–63.5%
**Gender**	
Male	117–65.7%
Female	61–34.3%
**Smoker**	
0	145–81.5%
1	33–18.5%
**Histology**	
Colon cancer	127–71.3%
Rectal cancer	51–28.7%
**Ras mutation**	
Ras *wild type*	103–57.9%
Ras mutated	63–35.4%
Unknown	12–6.7%
**M1 in organ sites**	
1	112–62.9%
≥2	66–37.1%
Treatment^*^	
F + O + TT	97–54.5%
F + I + TT	67–37.6%
F + TT	10–5.6%
F + O + I + TT	4–2.2%
**Initial body weight, BMI, SMI**	
**Variable**	**Total**
**Mean BW (kg)** (*n* = 178)	71.12 ± 15.9
Male	77.05 ± 14.3
Female	59.55 ± 10.78
**BMI groups** (*n* = 178)	
<18.5 kg/m^2^	7–3.9%
18.5–24.9 kg/m^2^	54–30.3%
25–29.9 kg/m^2^	53–29.8%
>30 kg/m^2^	33–18.5%
**Mean BMI (kg/m**^**2**^**)** (*n* = 178)	25.71 ± 4.71
Male	26.85 ± 4.72
Female	23.64 ± 3.97
**Mean SMI (cm**^**2**^**/m**^**2**^**)** (*n* = 81)	44.16 ± 10.80
Male (*n* = 51)	49.18 ± 9.63
Female (*n* = 30)	35.61 ± 6.46

* *F, Fluoropyrimidine; O, Oxaliplatin; I, Irinotecan; TT, Target therapy*.

### Outcomes and Toxicities

The outcomes varied depending on the treatment applied. In the-Pan treatment group the overall response rate (ORR) was 64.6%, the progression-free survival (PFS) was 13 months, and the OS, 21 months. In the ChT-Bev group, the ORR was 59.3%, PFS 10 months, and OS 19 months.

Any grade toxicities in the ChT-Pan treatment group were present in 58 (89.2%) patients. Severe toxicities (grade 3 and 4, CTCEA v5.0) were present in 26 (40%), mainly cutaneous in 14 (21.5%), hematological in 7 (10.8%), gastrointestinal in 4 (6.2%) and peripheral neuropathy in 3 (4.6%). Any grade toxicities in the ChT-Bev treatment group were present in 87 (77%) patients. Severe toxicities in 57 (50.4%), mainly hematological in 27 (23.9%), gastrointestinal in 15 (13.2%), and peripheral neuropathy in 6 (5.3%). The incidence of higher severe hematological and gastrointestinal toxicities in the ChT-Bev group compared with the ChT-Pan group may be, in part, explained by using a triplet chemotherapy protocol in 4 patients. DLT were present in 43 patients (66.2%) of the ChT-Pan group and in 66 (58.4%) in the ChT-Bev. Two toxic deaths were observed in the ChT-Bev group due to febrile neutropenia and none in the ChT-Pan treatment group.

### Factors Affecting Overall Survival and Dose Limiting Toxicities

**Figure 1:**
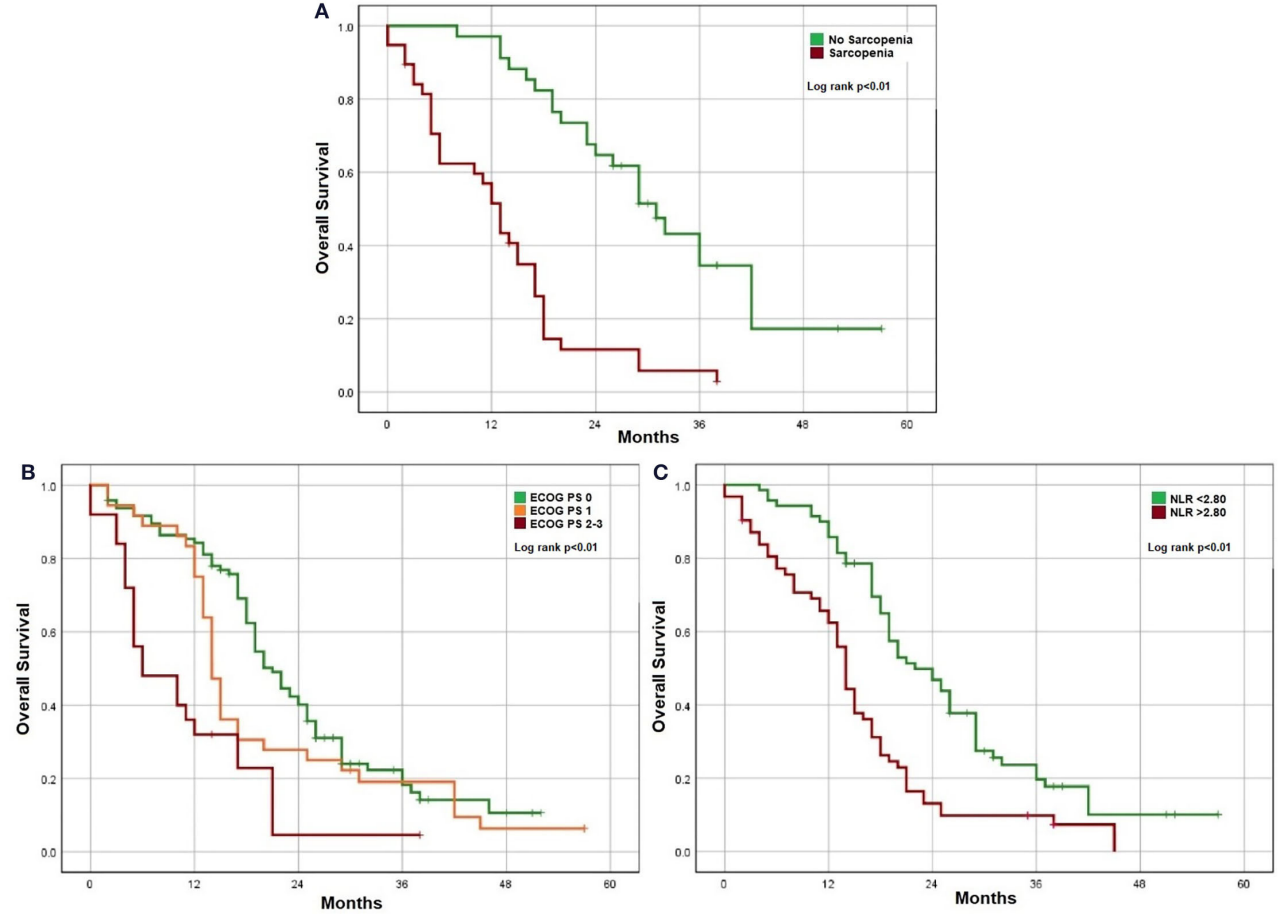
Factors associated with OS. **(A)** Sarcopenia. **(B)** ECOG performance status. **(C)** Systemic inflammation.

For the investigation of factors associated with OS and DLT, only unresectable mCRC patients were assessed (*n* = 157). Clinically relevant factors were analyzed in a univariate analysis and factors that demonstrate significant association (*p* < 0.05%) with this method were posteriorly analyzed in a multivariate model. In a multivariate analysis using a Cox regression model, higher ECOG PS, systemic inflammation with NLR > 2.80, and presence of sarcopenia were associated with lower OS (Figure 1 and Table 2). Using a logistic regression model, age and sarcopenia were associated with increased DLT. Patients with sarcopenia presented more DLT in comparison with patients without it (76.3 vs. 44.1%). Only 3 patients in this sample presented sarcopenic obesity, remarkably all of them presented DLT.

**Table 2:** Factors influencing OS and DLT.

**Variable**	***p*-value**	**Hazard ratio [IC 95%]**	***p*-value**	**Hazard ratio [IC 95%]**
	**Overall survival (OS)**		**OS – Multivariate analysis**	
	**Univariate analysis**		**Cox regression model**	
**Sarcopenia**	***p*** **<** **0.001**	HR 4.36 [2.45–7.74]	***p*** **=** **0.001**	HR 4.73 [1.85–12.09]
**NLR** **>** **2.80**	***p*** **<** **0.001**	HR 2.21 [1.52–3.22]	***p*** **=** **0.038**	HR 2.20 [1.05–4.62]
**ECOG PS**	***p*** **=** **0.004**		***p*** **=** **0.011**	
PS 0*		-		-
PS 1	NS (*p* = 0.119)	-	-	-
**PS** **≥2**	***p*** **<** **0.001**	HR 2.99 [1.85–4.83]	***p*** **=** **0.014**	HR 5.46 [1.42–21.10]
BMI	***p*** **<** **0.001**		NS *p* = (0.580)	
Underweight*		-		-
Normal	***p*** **=** **0.009**	HR 0.28 [0.11 - 0.73]	-	-
Overweight	***p*** **<** **0.001**	HR 0.18 [0.07 - 0.47]	-	-
Obesity	***p*** **<** **0.001**	HR 0.12 [0.04 - 0.32]	-	-
M1 in 1 or ≥ 2 sites	***p*** **=** **0.029**	HR 1.48 [1.04 - 2.09]	NS *p* = (0.132)	-
Ras mutation	NS (*p* = 0.076)	-	-	-
Age	NS (*p* = 0.169)	-	-	-
Colon laterality	NS (*p* = 0.322)	-	-	-
**Variable**	***p*-value**	**Odds ratio [CI 95%]**	***p*-value**	**Odds ratio [CI 95%]**
	**Dose limiting toxicities (DLT)**		**DLT—multivariate analysis**	
	**Univariate analysis**		**Logistic regression model**	
**Sarcopenia**	***p*** **<** **0.001**	OR 4.08 [1.48–11.19]	***P*** **=** **0.030**	HR 4.13 [1.15–14.80]
**Age**	***p*** **=** **0.033**	OR 1.04 [1.01–1.07]	***P*** **=** **0.014**	HR 1.09 [1.02–1.16]
NLR >2.80	***p*** **<** **0.001**	OR 3.76 [1.75–8.14]	NS (*p* = 0.145)	-
**ECOG PS**	***p*** **=** **0.046**		NS (*p* = 0.603)	-
PS 0*		-		
PS 1	NS (*p* = 0.236)	-		
PS ≥2	***p*** **=** **0.029**	OR 3.25 [1.13–9.36]		
Mean BMI	NS (*p* = 0.095)	-	-	-

*p-values and variables that are statistically significant in a multivariate analysis*.

## Discussion

**Figure 2:**
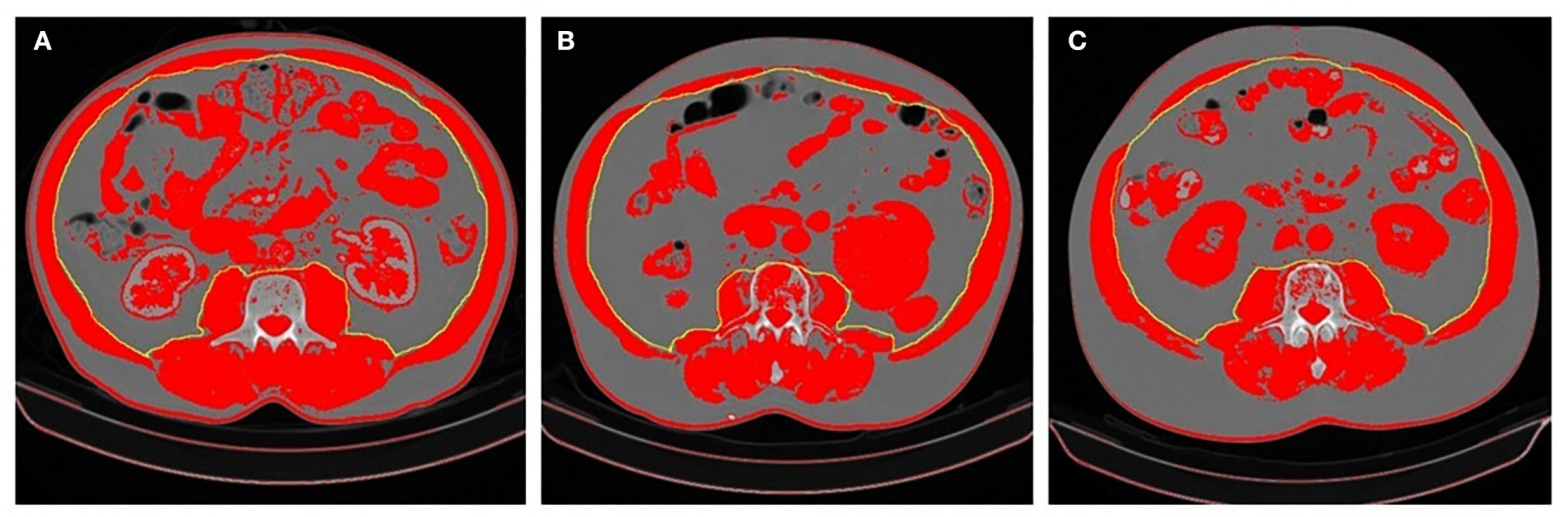
Differences in body composition in three patients. **(A)** Patient overwright (BMI 28.13 kg/m^2^) with no sarcopenia (SMI 62.80 cm^2^/m^2^); **(B)** Patient overwright (BMI 28.70 kg/m^2^) with sarcopenia (SMI 45.40 cm^2^/m^2^); **(C)** Patient with sarcopenic obesity (BMI 34.51 kg/m^2^ and SMI 46.10 cm^2^/m^2^). Patients **(B,C)** presented DLT. The areas in red represent values between −29 and +150 HU. The SMA was determined based on the area of the *psoas major, quadratus lumborum, erector spinae, latissimus dorsi, abdominal oblique muscles*, and *rectus abdominis muscles*. SMI was calculated by the SMA/height^2^.

Body composition evaluation by CT scan could be performed in patients with cancer, as they undergo this exam routinely at diagnosis and follow-up. The most used software in the literature is the commercially available and validated *Slice-O-Matic. NIH ImageJ* software appears to have the same precision detecting SMA (17). This method, although it can be performed in other cross-section regions, is usually performed at the cross-section of the L3 vertebra, as there is a close correlation between muscle and fat areas and corresponding tissue volumes, as such, it is considered a reference point for both sexes (18). There are no established cut-off values to define sarcopenia with this method. Some studies apply the cut-off used in Prado et al. study (19), defined as SMI < 52.40 cm^2^/m^2^ for men and < 38.50 cm^2^/m^2^ for women. In Asian patients, the cut-off are usually lower as < 36 cm^2^/m^2^ for men and < 29 cm^2^/m^2^ for women (20). The authors believe these cut-off values presented have some degree of heterogeneity, including population with different tumors, with different biological activity as well as different tumor staging. Similarly, patients with cancer living in different locations worldwide, present different body composition, as seen in the above examples. This heterogeneity is a major hindrance in the implementation of this method in clinical practice. In this study, the cut-off values for sarcopenia for this specific Portuguese population with mCRC were SMI < 49.12 cm^2^/m^2^ for men and < 35.85 cm^2^/m^2^ for women. The prevalence of sarcopenia was 49.4% while the mean BMI was 25.71 kg/m^2^, which suggests that patients may be sarcopenic even though they don't present visible signs of malnutrition (21, 22). An example can be observed in Figure 2.

In this study, sarcopenic patients showed worse OS, in line with the consistent growing evidence of the prognostic value of this method (23–25). It was also able to predict DLT, which goes in accordance to the literature (21, 26), although there is still some debate on this matter, as in the case of CAIRO 3 population study, sarcopenia at diagnosis was not predictive of DLT, only a loss > 2% of SMI in the 3 months follow up was a predictor of DLT (22). Sarcopenic obesity is a specific entity, associated with several health-related risks, particularly related to increased toxicities and reduced OS in oncological patients. In this study only 3 patients presented sarcopenic obesity, interestingly all of them developed DLT (19, 27). It raises the question of whether the conventional body surface area is the most appropriate method of dosing cytotoxic drugs. A hypothesis for the excess of toxicity in this population suggests that many ChT drugs are hydrophilic and consequently, patients receive a higher absolute dose while presenting a reduced volume of distribution, leading to higher dose concentrations and increased toxicities (27, 28). Sarcopenia identified by CT image at the L3 region appears to be more precise than anthropometric measurements for prognosis and prediction of DLT in patients with mCRC.

Interventions in a late phase of the disease usually are refractory to any treatment modality, therefore, early identification of sarcopenic patients is of utmost importance to elaborate a personalized multimodal approach with nutritional intervention and a physical exercise plan. This approach should be based on disease burden, intent of treatment, patient's physical performance, and desires (29). This personalized approach is crucial, considering that Portuguese patients with mCRC usually present insufficient mean caloric and protein intake, 20.1 Kcal/kg and 0.85 g/kg respectively, much lower than the recommendations of 25–30 Kcal/kg and 1.5g/kg by ESPEN (30, 31).

As a retrospective study, several limitations are present and should be considered as it relies on data not primarily meant for research. Missing data were present and treated accordingly, some patients had no CT scan images in our institution database as they were performed in an external institution, which may introduce selection bias.

## Conclusion

Sarcopenia identified by CT evaluation at the L3 region was associated with significantly worse prognosis and increased dose-limiting toxicities in mCRC. The establishment of optimal cut-off values is still a barrier to implementing this method in clinical practice, and as such, studies with less heterogeneity should be conducted. Half the patients with mCRC presented sarcopenia, and an early introduction of a multimodal strategy to prevent muscle waste should be considered.

## Data Availability Statement

The raw data supporting the conclusions of this article will be made available by the authors, without undue reservation.

## Ethics Statement

The studies involving human participants were reviewed and approved by Comissão de Ética para a Saúde. Written informed consent for participation was not required for this study in accordance with the national legislation and the institutional requirements.

## Author Contributions

DD performed the literature research, the statistical analysis, the interpretation of data, and wrote the draft of this manuscript. MM extracted L3-CT scan images and added valuable information. CT critically reviewed the document and added valuable information. BG critically reviewed the document and added valuable information. PR critically reviewed the document and added valuable information. All authors have read and agreed to the published version of the manuscript.

## Conflict of Interest

The authors declare that the research was conducted in the absence of any commercial or financial relationships that could be construed as a potential conflict of interest.

## Publisher's Note

All claims expressed in this article are solely those of the authors and do not necessarily represent those of their affiliated organizations, or those of the publisher, the editors and the reviewers. Any product that may be evaluated in this article, or claim that may be made by its manufacturer, is not guaranteed or endorsed by the publisher.
