# Corrigendum: Impact of body composition on prognosis and dose-limiting toxicities on metastatic colorectal cancer

**DOI:** 10.3389/fnut.2023.1252840

**Published:** 2023-07-25

**Authors:** David da Silva Dias, Mafalda Machado, Carolina Trabulo, Beatriz Gosálbez, Paula Ravasco

**Affiliations:** ^1^Medical Oncology Department, Centro Hospitalar Universitário do Algarve, Faro, Portugal; ^2^Centre for Interdisciplinary Research in Health, Universidade Católica Portuguesa, Lisbon, Portugal; ^3^Faculdade de Ciências da Saúde da Universidade da Beira Interior, Covilhã, Portugal; ^4^Radiology Department, Centro Hospitalar Universitário do Algarve, Faro, Portugal; ^5^Medical Oncology Department, Centro Hospitalar Barreiro Montijo, Barreiro, Portugal; ^6^Católica Medical School, Universidade Católica Portuguesa, Lisbon, Portugal; ^7^Centro de Investigação Interdisciplinar Egas Moniz (CiiEM), Instituto Universitário Egas Moniz (IUEM), Caparica, Portugal

**Keywords:** body composition, body mass index, skeletal muscle index, sarcopenia, metastatic colorectal cancer, dose limiting toxicities, neutrophil-to-lymphocyte ratio, systemic inflammation

In the published article, there was an error regarding the affiliation for David da Silva Dias. As well as having affiliations 1 and 2, they should also be affiliated to “Faculdade de Ciências da Saúde da Universidade da Beira Interior, Covilhã, Portugal.”

The authors apologize for this error and state that this does not change the scientific conclusions of the article in any way. The original article has been updated.

